# Parental Knowledge on car Safety for Children: An Israeli Survey

**DOI:** 10.1100/tsw.2006.04

**Published:** 2006-01-17

**Authors:** Michal Hemmo-Lotem, Jacob Urkin, Liri Endy-Findling, Joav Merrick

**Affiliations:** ^1^ Beterem National Center for Child Safety and Health, Petach Tiqva, Israel; ^2^ School of Public Health, Haifa University, Haifa, Israel; ^3^ Pediatric Primary Care Unit, Ben-Gurion University of the Negev, Beer-Sheva, Israel; ^4^ Faculty of Health Sciences, Ben-Gurion University of the Negev, Beer-Sheva, Israel; ^5^ Division of Pediatrics, Soroka University Medical Center, Beer-Sheva, Israel; ^6^ National Institute of Child Health and Human Development, Faculty of Health Sciences, Ben Gurion University of the Negev, Beer-Sheva, Israel; ^7^ Center for Multidisciplinary Research in Aging, Faculty of Health Sciences, Ben Gurion University of the Negev, Beer-Sheva, Israel; ^8^ Office of the Medical Director, Division for Mental Retardation, Ministry of Social Affairs, Jerusalem, Israel

**Keywords:** car safety, parents, children, public health, Israel

## Abstract

The objective of this study was to assess the level of parental car safety knowledge and actual behavior regarding their children under the age of 15 years. This study forms part of the National Center for Child Safety and Health in Israel (Beterem) program to examine awareness on child safety. Seven hundred and five Jewish families with at least one child at home younger than 15 years (a total of 1,345 children) were used as a randomized sample of the Jewish population. A telephone survey was conducted by professional interviewers using a questionnaire developed by injury prevention specialists consisting of seven knowledge questions and a diagram that described the usual seating positions and restraining method of the family members in the family car. Concerning knowledge about injury prevention, the rate of incorrect answers was high,64% in regard to the proper car seats for age and 84% in regard to the age for booster seats. Sixty five per cent of parents did not know what a booster seat was and 54% did not know that the proper place for children was in the back seat. The average of incorrect answers was 4.86 out of 7 (SD=1.45) correlated with low socioeconomic status. Concerning care safety behavior 60% per cent of babies and 38% of toddlers were not restrained properly. This study should alert planners and policy makers to the need of implementation of educational prevention programs for the Israeli public of parents concerning car safety for children in order to reduce childhood injury.

## INTRODUCTION

As in most industrialized countries, injuries in Israel are the primary cause of mortality in children and youth with loss of potential years of life[1]. Motor vehicle accidents were the most common cause of injury related death (3:100,000), drowning (1:100,000), while falls, burns, suffocation and poisoning (less than 1:100,000 each) were less frequent causes[2]. Death is the most severe outcome of an injury, but each injury must be seen as a public health concern as it usually results in an emergency room visit, hospitalization and transient, or permanent, disability.

In a survey from 1994[2], the rate of pediatric injury-related visits to emergency departments in Israel was 712 per 10,000, the hospitalization rate was 67 per 10,000 and the death rate was 8 per 10,000. In the year 1999 alone, 206,583 injured children visited the pediatric emergency departments of the major 24 hospitals in Israel[3]. Between the years 1994 and 1999, an increase in the proportion of children who visited the pediatric emergency department due to injury was noted[3]. The highest rates of injuries were encountered in the 1-4 and 15-17 year olds[3]. Between 30-50% of pediatric emergency department visits were injury related and ten per cent of the children were hospitalized. One out of 90 hospitalized children with injuries died[2,3].

The hospitalization rate of injured children aged 1-4 years was the highest[2,3]. Of all the hospitalizations due to unintentional injuries, the most common were falls (43%), road accidents (24%), blunt trauma (9%) and burns (7%)[2,3]. Less frequent causes for hospitalization were poisoning (4%), stings and bites (4%), sharp objects (3%), foreign bodies (2%) and other (4%)[2,3]. The main reasons for pediatric emergency department visits related to unintentional injuries: falls (41%), contusion (19%), road accident (15%), foreign body (5%), sharp objects (5%), stings and bites (3%), burn (3%), poisoning (1%) and other (7%)[2,3].

The impact of injuries is enormous including short and long term disabilities, suffering and the economic burden on society[4,5]. Seventy one per cent of injuries are preventable by the use of simple means[6,7]. Their implementation resulted in 35% reduction of injury related mortality over a period of ten years[8].

According to the model of William Haddon, Jr. (1926-1985), the founding father of injury prevention research, injury is a result of interaction between the person, the product, the environment and the socioeconomic milieu[9]. By proper intervention on these four components, injury can be prevented, or minimized, before, at the time of, or following the event. Some of the methods which can help to focus on high risk groups include epidemiology studies on the age, gender, socioeconomic and geographic background of the injured. Such thinking can lead to interventions that reduce childhood injuries, but in order to be effective it should be translated into a change toward a safer behavior and environment. Combining more than one strategy makes the intervention more effective, such as: legislation, law enforcement, environmental changes, product changes, education and change of public awareness[4,5,7,8].

The effectiveness of passenger restraint in cars on reducing injuries has been well documented[10]. Car safety seats reduce automobile related deaths by 71% in babies and by 54% in infants. Improperly restraining children by not using booster seats increased the risk of head injury by four fold[11]. A survey done in 1995 indicated that only 64% of children up to the age of 12 years were restrained in cars[12]. Restraining was less frequent as the child's age increased, even though parents knew the rules and recommendations, which was also shown by a study in Baltimore[11]. The first national telephone survey on child safety conducted in the United States indicated that parents had more concern about child abduction and street-drugs than childhood injuries, with lack of knowledge related to low socioeconomic status[13].

The aim of the present study was to conduct a survey on the knowledge of Israeli parents of children up to 15 years of age about car safety and their behavior regarding their children.

## METHODS

Seven hundred and five Jewish families with at least one child at home younger than 15 years old (a total of 1,345 children) were selected as a randomized sample of stratification of the Jewish population who resided in cities and towns with more than 20,000 inhabitants.

Professional interviewers trained in the study forms, in order to accomplish uniformity and validity, conducted a telephone survey. The interviews were conducted between 4 and 10 PM (16:00-22:00) in the second half of the month May. Quality assurance measures were implemented. The response rate was 31% of the phone calls.

Injury prevention specialists developed the questionnaire. It was validated and a pilot study was conducted on a sub-sample of the target population. The questionnaire consisted of seven knowledge questions and a diagram that described the usual seating positions and restraining methods of the family members in the family car.

## RESULTS

The rate of incorrect answers was high for all questions, 64% in regard to the proper car seats for age and 84% in regard to the age for booster seats. Sixty five per cent of parents did not know what a booster seat was and 54% did not know that the proper place for children was in the back seat. Eighty three per cent did not know that infants in rear-facing safety seats on the passenger side can be severely injured because their heads are in the direct path of an inflating air bag. Ninety nine per cent of the parents were mistaken regarding the proper facing of a baby. Only one percent of parents knew all the right answers. The average of incorrect answers was 4.86 out of 7 (SD=1.45). Parents who did not graduate high school and those of low socioeconomic status and religious orthodoxy demonstrated the lowest knowledge scores.

Parents were asked to describe a typical family ride in their private car. The position of each family member in the car and the use of restraining methods for each were described. Sixty per cent of babies and 38% of toddlers were not restrained properly. Ninety six per cent of children between the age of 4 to 10 year old and 22 % of the age 10 to 14 years were not restrained properly. The use of an age-appropriate restraining device was 40% for babies, 62% for toddlers, 4% for children aged 4 to 10 years and 78% for the 10 to 14 years old. Almost all parents were properly restrained.

For babies (0 to one year old), there was a significant relationship between knowledge and alleged behavior ($\chi^2$=14.23, p<.05). Sixty nine per cent of parents who restrained their babies properly demonstrated good knowledge as opposed to 38% of the parents who did not restrain their babies properly. Sixty seven per cent of the parents who did not restrain babies facing the rear of the car and 47% of those who did, did not know that the back seat was the right place for the baby's car seat ($\chi^2$ =6.53, p<.05).

For toddlers and children aged 4-10 years, there was also a significant relationship between knowledge and alleged behavior ($\chi^2$=7.89, p<.05) ($\chi^2$=4.33, p<0.05) respectively. Seventy eight per cent of parents who restrained their toddlers properly demonstrated good knowledge as opposed to 50% of the parents who did not restrain their babies properly. For children aged 4-10 years, the rates were 38% and 12% respectively. Only 9.8% of parents of toddlers knew what a booster seat was. Forty nine per cent of parents who restrained their toddlers properly did not know about the booster as the recommended seat for children between 4 to 10 years old. Eighty seven per cent of parents who use booster seats for children of 4 to 10 year old knew what a booster seat was. Just 41% of the parents who restrained children of 4 to 10 year old with a belt only knew about booster seats.

## DISCUSSION

The level of parental knowledge regarding restraining children in cars and their alleged behavior is a major public health concern. It seems that too many parents lack the knowledge that a baby up to one year of age should be restrained in the back seat facing backwards. Many parents do not know that a safety belt without a booster seat is not safe enough for children aged 4 to 10 years. Parents are therefore exposing their children to unnecessary risks without even knowing it. Children in the greatest risk are from families of low socioeconomic status, as poor knowledge about car safety and inappropriate behavior are related to poverty and parental level of education[13].

Educating parents about car safety should focus on the recommended positioning of children in the car, on the appropriate facing of the child and the age-appropriate restraining device with age 4 years as the right time to change from baby seat to a booster seat. Non-adherence to this recommendation is endangering the child, as the car baby seat is too small to restrain a toddler.

The target population for education about car safety for children is parents and those with babies should be aimed at in particular. Babies are completely passive in regard to safe behavior and thus totally dependent on their parents' behavior. Should a car accident happen while they are not properly restrained, they are at greater risk to encounter a head injury due to their size and elevated center of gravity.

Efforts to improve the behavior of parents should include many modalities. It should start with educating parents who are going to have a baby. It should be emphasized in each visit to the well baby clinic. The primary care health worker should advocate car safety on each encounter. Efforts should include updating these professionals with the current recommendations. Whenever possible, the shopping centers where parents are buying goods for their children, should add advertisements on safety devices and safe behavior. These can be also done on products like baby food, toys and decorations. The media should be used to inform the public and to lower tolerance of inappropriate behavior. School aged children should be educated at schools about car safety and serve as safety advocates in their homes.

Legislation on car safety and law enforcement should supplement other modes of intervention. Only combined action over an extended period of time can make the necessary change in parental behavior. The effect of such educational programs should be reflected in the reduction of car related injuries for children, since it should be possible to reach a higher level of preventable childhood injury[14].

This study has limitations due to being a telephone survey with a low response rate.

## BIOSKETCHES

**Michal Hemmo-Lotem, MD**, is a pediatrician and a graduate of the School of Educational Leadership in Jerusalem. She has established Beterem-The National Center for Children's Health and Safety in Israel and currently the director (CEO). Beterem dedicates its work to childhood injury prevention, safety and research. She is the chairman of the National Committee for Childhood Injury Prevention and Safety and member of the National Council for Child Health and Pediatrics, the National Council for Health Promotion and involved in the public debate on childhood injury and safety. She is lecturer at the School of Public Health, Haifa University. E-mail: mhemmo@beterem.org Website: http://www.beterem.org/Pages/english.asp

**Jacob Urkin MD, MPH**, is a lecturer in pediatrics and director of the Primary Pediatric Unit and director of the Maternal and Child program of the MPH program at the Faculty of Health Sciences, Ben Gurion University of the Negev, Beer-Sheva, Israel. Major academic interests are: primary care, community pediatrics, well baby care services, school health and medical education. E-mail: jacobur@clalit.org.il

**Liri Endy-Findling, MPH** educated at the School of Public Health at the Hebrew University is the Director of the Department of Research, Development and Policy at Beterem-the National Center for Children's Health and Safety, Israel. Beterem is an organization focused on injury prevention and safety promotion in childhood and Liri Endy-Findling is one of the leading figures in the organization. She has a rich background as a researcher from her experience at the National Research Institute for Social Sciences and Beterem. She leads public policy, programs development, research management, promotion of legislation and standards in the field of safety. E-mail: liri@beterem.org.il

**Joav Merrick, MD, DMSc,** is professor of child health and human development affiliated with the Center for Multidisciplinary Research in Aging, Zusman Child Development Center, Division of Pediatrics and Community Health at the Ben Gurion University, Beer-Sheva, Israel, the medical director of the Division for Mental Retardation, Ministry of Social Affairs, Jerusalem, the founder and director of the National Institute of Child Health and Human Development. He has written numerous publications in the field of child health and human development, rehabilitation, intellectual disability, disability, health, welfare, abuse, advocacy, quality of life and prevention and has received the Peter Sabroe Child Award for outstanding work on behalf of Danish Children in 1985 and the International LEGO-Prize ("The Children's Nobel Prize") for an extraordinary contribution towards improvement in child welfare and well-being in 1987. E-Mail: jmerrick@internet-zahav.net. Website: www.nichd-israel.com
